# Effects of silencing RIP1 with siRNA on the biological behavior of the LoVo human colon cancer cell line

**DOI:** 10.3892/ol.2014.2040

**Published:** 2014-04-07

**Authors:** HONG-XIA YOU, YAN-HONG ZHOU, SHI-YUN TAN, TONG-HUI SHE

**Affiliations:** 1 Department of Gastroenterology, Hubei University of Science and Technology, Xianning, Hubei 437100, P.R. China; 2 Department of Gastroenterology, Renmin Hospital of Wuhan University, Wuhan, Hubei 430060, P.R. China

**Keywords:** RNA interference, RIP1 gene, colorectal carcinoma

## Abstract

The present study aimed to investigate the effects of silencing RIP1 by small interfering RNA (siRNA) on the biological behavior of the LoVo human colorectal carcinoma cell line and to provide evidence for the feasibility of colorectal cancer gene therapy. LoVo cells were divided into the RIP1 siRNA group, the blank control group and the negative control group. Chemically synthesized siRNA targeting RIP1 (RIP1 siRNA) was transfected into LoVo cells. Following transfection of the RIP1-targeted siRNA into the LoVo cells, the expression of the RIP1 gene was effectively inhibited. The results demonstrated that RIP1 effectively regulated the malignant biological behavior of the LoVo colon cancer cell line. Furthermore, the proliferation, motility and invasiveness of LoVo cells were inhibited by siRNA knockdown of RIP1. The results revealed that the RIP1 gene has an important role in the regulation of proliferation and apoptosis in colorectal carcinoma cells.

## Introduction

Colorectal cancer (CRC) is a highly prevalent cancer in males and females, which is associated with a severe demographic and economic burden worldwide. In the United Kingdom, one in every 20 people will be affected by CRC, which is often well established and metastatic beyond the bowel at first diagnosis (1). Despite efforts to improve early detection, the precise mechanisms underlying the malignant consequences of colorectal cancer remain unclear. Inflammatory syndromes, hereditary factors and environmental factors are all risk factors associated with CRC (2). For the last two decades, the liver has been the most frequent site of metastasis of colorectal cancer (3), which affects the efficacy of treatment and the prognosis of patients.

The receptor-interacting protein (RIP, RIPK) is widely expressed in the majority of cell types. RIP is a family of kinases that mediates cellular survival as well as inflammatory and immune responses (4). There are seven RIP proteins (RIP1–RIP7). The RIPs are 74-kDa proteins that contain an N-terminal region and a C-terminal region that includes an intracellular death domain (5). RIP1 is the most important member of the RIPs, and it mediates mitochondrial dysfunction, ROS and activation of the transcription factor NF-κB, and is crucial for programmed necrosis. RIP1 is required for the activities of TLR3 and TNF-α, as well as DNA damage (6–8). RIP1 ultimately targets cellular energetic metabolism and autophagy through mechanisms related to innate and adaptive immunity (9). If ubiquitination of RIP1 is blocked, necrosis ensues when caspases are inhibited. Recruitment of NEMO to ubiquitinated RIP1 is important for the TNFR1 signaling pathway, which determines whether RIP1 triggers a necrotic death response (10). RIP1-deficient mice appear normal at birth, but apoptosis occurs in the lymphoid and adipose tissues, and the mice die at 1–3 days old (11). RIP1 inhibits caspase-8 cleavage and TRAIL-induced apoptosis (12).

The association between RIP1 and colorectal cancer has been previously studied. These studies revealed that RIP1 affects cellular proliferation, apoptosis, angiogenesis and cell adhesion in tumor cells (13).

In the present study, RNA interference (RNAi) was utilized with a HiGene to target RIP1 in colon cancer cells. RNAi, which is mediated by double-stranded RNA, is a sequence-specific and post-transcriptional gene silencing process. RNAi has become a highly useful tool for functional genomics and represents a novel therapeutic approach for treating human diseases that involve viral infections (14). Currently, RNA-based therapeutics that have the ability to silence the expression of specific targets are used for the clinical investigation of numerous disorders, including cancer. As the mechanisms of tumor evasion from the host immune system are versatile, to enhance the immune response against tumors, RNAi technology is used to target numerous different molecules (15). In the present study, RIP1 small interfering RNA (siRNA) was employed to knockdown the expression of RIP1 and its effects on cell proliferation, migration, apoptosis, the cell cycle, invasion and the related mechanisms were investigated.

## Materials and methods

### Materials

The LoVo human colon cancer cell line was obtained from the Cell Collection of Chinese Academy of Sciences (Shanghai, China). The transfections were performed using HiGene (Applygen, Beijing, China). The Annexin V-FITC/PI Apoptosis kit was obtained from BioVision (Mountain View, CA, USA). The BD Matrigel™ Basement Membrane matrix was purchased from BD Biosciences (San Jose, CA, USA). Transwell plates were obtained from Corning, Inc.(Corning, NY, USA) and 3-(4,5-dimethylthiazol-2-yl)-2, 5-diphenyltetrazoliumbromide (MTT) was purchased from Wuhan Kerui Biological Technology Co., Ltd. (Hubei, China). TRIzol reagent was obtained from Invitrogen Life Technologies (Carlsbad, CA, USA). The Fermentas kit was purchased from MBI Fermentas (Amherst, NY, USA). The 2× Taq PCR MasterMix was obtained from Tiangen Biotech Co., Ltd. (Beijing, China). Primers for human RIP1 and β-actin were designed by Sangon Biotech Co., Ltd. (Shanghai, China), and the sequences were as follows: Forward, 5′-GTC AAA TTC AGC CAC AGA ACA GCC-3′ and reverse, 5′-CCC TTT AG CCT TCC CTC ATC ACC-3′ for RIP1, 579 bp; forward, 5′-UUC UCC GAA CGU CUC ACG UTT-3′ and reverse, 5′-ACG UGA CAC GUU CGG ACA ATT-3′ for β-actin, 479 bp. RIP1 siRNA was purchased from Shanghai GenePharma Co., Ltd (Shanghai, China). The primary antibody was obtained from Beijing Biosynthesis Biotechnology Co., Ltd (Beijing, China). The colon cancer tissue microarray was purchased from Fanpu Biotech, Inc. (Guiling, China). The study was approved by the ethics committee of Hubei University of Science and Technology (Xianning, China).

### Cell culture

LoVo cells were maintained in RPMI-1640 culture medium (Gibco Invitrogen Corporation, Grand Island, NY, USA) supplemented with 10% fetal bovine serum (FBS; Hyclone, Logan, UT, USA), 1% penicillin/streptomycin (Beyotime Institute of Biotechnology, Haimen, China) and 2 mM L-glutamine (Amresco LLC, Solon, OH, USA). The cells were grown in monolayer cultures with 5% CO_2_ in a humidified 37°C incubator. Every 2–3 days, the cells were subcultured. When the cells reached the logarithmic growth phase, 0.25% trypsin (Amresco LLC) was applied for 1–3 min. The cells were resuspended in RPMI-1640 containing 10% FBS at a cell concentration of 1×10^4^ cells/ml.

### RIP1 siRNA synthesis and transfection

RIP1 siRNA was synthesized by Shanghai GenePharma Co., Ltd (Shanghai, China). RIP1 siRNA was modified with 5-FAM (Shanghai GenePharma Co., Ltd) fluorescence by targeting positions (siRNA1: RIP1-Homo-654 forward, 5′-GGG CGA UAU UUG CAA AUA ATT-3′ and reverse, 5′-UUA UUU GCA AAU AUC GCC CTT-3′; siRNA2: RIP1-Homo-1701 forward, 5′-GCC AGC UGC UAA GUA CCA ATT-3′ and reverse, 5′-UUG GUA CUU AGC AGC UGG CTT-3′) of the RIP1 transcript (GenBank accession no., NM_003804.3). Simultaneously, a random negative siRNA was utilized as a negative control. The cells were divided into three groups, which included a blank control group supplemented with only the transfection reagent, a negative control group (LoVo-Con) containing a non-targeting control siRNA and transfection reagent, and the experimental group (LoVo-RNAi) containing RIP1 siRNA and the transfection reagent. For cell transfection, the cells were plated on six-well plates (4×10^5^ cells for LoVo) in RPMI-1640 with 10% FBS. After 12 h, 30 μl siRNA per well was incubated with HiGene and 9.6 μl of the mixture (30 μl siRNA and 9.6 μl HiGene) was added per well according to the manufacturer’s instructions. The transfected cells were incubated in a humidified 37°C incubator with 5% CO_2_ for ~24 h.

### Examination of morphological changes

Following the application of LoVo-Con siRNA or RIP1 siRNA for 24 h, an inverted phase-contrast microscope (Nikon, Tokyo, Japan) was utilized to observe the morphological changes in the cells. Images were captured using a digital camera (Nikon) at a magnification of ×200.

### Cell cytotoxicity assay

The MTT assay was used to evaluate the LoVo cell viability. In brief, LoVo cells in the logarithmic growth phase were digested with 0.25% trypsin and the cell concentration was adjusted to 0.5×10^5^ cells/ml. The LoVo cells were seeded in 96-well plates at a density of 200 μl/well. The cell suspension (200 μl) was divided into three groups. The blank control group included the transfection reagent and LoVo cells; the negative control group (LoVo-Con) included a non-targeting control siRNA, the transfection reagent and LoVo cells; and the experimental group (LoVo-RNAi) included the RIP1 siRNA, the transfection reagent and LoVo cells. The three groups were incubated in a humidified 37°C incubator with 5% CO_2_ for 24, 48 and 72 h. Then, 20 μl of 5 mg/ml MTT solution was added to each well and the cells were incubated at 37°C in a humidified incubator with 5% CO_2_ for ~4 h. When the media was discarded, 200 μl DMSO was added to each well. The optical density at 490 nm was read using an iMark Microplate Absorbance reader (Bio-Rad, Hercules, CA, USA). The results [the optical density, (OD)] are presented as the mean ± standard deviation (SD). The cell proliferation inhibitory rate was calculated as follows: Cell proliferation inhibitory rate (%) = (1-A_490_ of experimental well/A_490_ of blank control well).

### Cell cycle analysis

For flow cytometry (FCM) cell cycle analysis, to measure the cellular DNA content, the LoVo cells, negative siRNA-transfected cells and the RIP1 siRNA-transfected cells (LoVo, LoVo-Con and LoVo-RNAi) were centrifuged at 800 × g for 5 min, collected, washed with ice-cold phosphate-buffered saline (PBS) and fixed in cold 70% ethanol at 4°C until staining. Then, the cells were centrifuged at 800 × g for 5 min, washed with PBS twice and incubated with 500 μl RNase A (100 μg/ml) and propidium iodide [PI; 0.2% in Triton X-100 (Jianglai Biotech, Inc., Shanghai, China)] purchased from (Sigma-Aldrich, St. Louis, MO, USA) for 30 min at 4°C in the dark. The cell cycle was detected using the Annexin V-FITC Apoptosis Detection kit. The cells (~20,000–30,000) were counted and the ModFit cell cycle fitting software (Verity Software House, Inc., Topsham, ME, USA) was used for the analysis. The experiments were repeated three times.

### Matrigel invasion assay

Transwell chambers (Corning, Inc.) were used to perform the cell invasion assay. Following 24 h of transfection, 2×10^4^ cells were suspended in 200 μl of serum-free medium and inoculated into each ECM-coated upper compartment of the 24-well plates that were precoated with 50 μl of 1 μg/μl Matrigel (BD Biosciences). The lower compartment of each chamber was suspended in 10,000 μl RPMI-1640 with 30% fetal calf serum and incubated for 48 h at 37°C with 5% CO_2_. Then, the cells on the upper surface were removed using cotton tips and the cells that migrated to the underside of the membrane were fixed with methanol for 30 min, stained with 0.1% crystal violet (Tianjin Yixin Hengxin Chemical Co., Ltd., Tianjin, China) for 30 min and washed with PBS three times. The number of tumor cells was calculated in five random fields at a magnification of ×200, using an inverted microscope (DP70, Olympus, Tokyo, Japan) and expressed as the average number of cells/field of view.

### Cell counting assay

When the cells were in the logarithmic growth phase, 0.25% trypsin was used to dissociate the cells and the cells were counted in a suspension with RPMI-1640 medium containing 10% FBS. Based on the cell counting results, the cells were cultured at a density of 4×10^5^/well in six-well plates. At this stage, two additional six-well plates were inoculated. After ~24 h, the cultures were divided into three groups (two wells in each group: the normal group, negative control group and the experimental group). The three six-well plates were cultured for 24, 48 and 72 h, respectively. A cell counting board was used to count and calculate the average number of cells in each group. The experiments were repeated three times. According to the results of cell counting, growth curves were generated with the number of unit cells (cells/ml) as the longitudinal coordinates and time as the abscissa.

### Immunohistochemistry

The rabbit anti-human RIP1 polyclonal antibody was purchased from Beijing Biosynthesis Biotechnology Co., Ltd. (Beijing, China). The colorectal carcinoma chip was purchased from Fanpu Biotech, Inc. (China). This chip included 36 patients with different types of colon tumor and samples of normal and inflammatory tissues from 12 patients. The 36 colon tumors included three cases of adenocarcinoma with pathological grade I, ten cases of adenocarcinoma with pathological grade I–II, nine cases of adenocarcinoma with pathological grade II, three cases of adenocarcinoma with pathological grade II–III, five cases of adenocarcinoma with pathological grade III, two cases of papillary adenocarcinoma, four cases of mucinous adenocarcinoma, one case of stromal tumor and one case of lymphoma. The tissues were cut into 4-μm-thick slices. The immunohistochemical SP method was performed according to the manufacturer’s instructions, with known positive tissue sections as a positive control and PBS instead of primary antibody as a negative control. The procedure was conducted as follows: i) dewaxing and hydration; ii) antigen repair; iii) incubation with normal goat serum solution at 37°C for 10 min; iv) application of the primary antibody at 4°C and incubation overnight followed by washing with PBS for 3 min, incubation with the biotin-labeled secondary antibody at 37°C for 30 min and washing with PBS for 3 min; v) application of the horseradish peroxidase-labeled streptomycin avidin working solution and washing with PBS for 3 min; vi) DAB/H_2_O_2_ reactive dyeing followed by thorough rinsing with water, hematoxylin staining, dehydration, transparent, drying and mounted with a neutral gum.

### Quantitative (q)PCR assays

Total RNA was isolated from the LoVo human colon cancer cell line using TRIzol reagent (Invitrogen Life Technologies). cDNA was synthesized using 2× Taq PCR MasterMix (Tiangen Biotech Co., Ltd.). A 25 μl reaction mixture was composed containing 1 μl cDNA, 2 μl of the upstream and downstream primers (MBI Fermentas, Glen Burnie, MD, USA), 12.5 μl PCR-2× master mix (Tiangen Biotech Co., Ltd.) and 9.5 μl RNase-free water (Amresco LLC). PCR was executed according to the manufacturer’s instructions. The cycling conditions included a holding step at 94°C for 3 min and 29 cycles of 94°C for 30 sec, 55°C for 30 sec and 72°C for 1 min. Relative quantification was analyzed using the ΔΔCT method.

### Statistical analysis

The statistical software package SPSS 17.0 was used for statistical analysis (SPSS, Inc., Chicago, IL, USA). The experimental results are presented as the mean ± SD. Data analysis was performed with Student’s t-test and the χ^2^ test. P<0.05 was considered to indicate a statistically significant difference.

## Results

### Fluorescent detection of transfected cells

**Figure 1:**
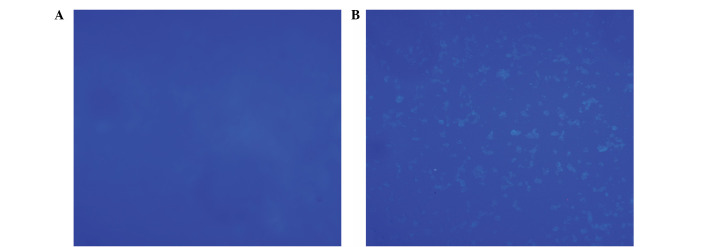
Fluorescent detection of transfected cells. The cells were transfected with receptor-interacting protein 1 small interfering RNA for 24 h. (A) LoVo cells were observed under a fluorescence microscope. (B) The LoVo-RNA interference group was observed under a fluorescence microscope with FAM-labeled green fluorescence.

Following transfection of LoVo cells with RIP1 siRNA (0.1 μg/μl) for 24 h, the fluorescence was observed under a fluorescence microscope. Compared with the colon cancer cells, the colorectal cancer cells that had been transfected with RIP1 siRNA exhibited a green fluorescence (Fig. 1). The results confirmed than RIP1 siRNA had been transfected into the LoVo cells.

### Effect of RIP1 siRNA on the morphology of LoVo cells

**Figure 2:**
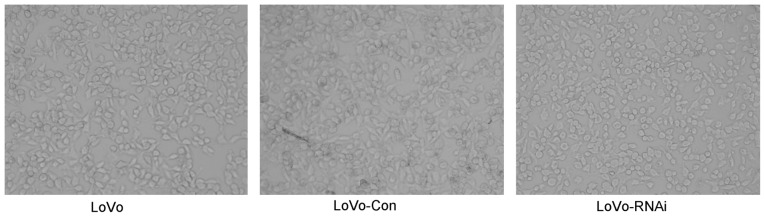
Effect of RIP1 siRNA on the morphological changes of LoVo cells. Following application of the negative control (LoVo-Con) siRNA or RIP1 siRNA (LoVo-RNAi) for ~24 h, the morphological changes were observed with an inverted phase-contrast microscope. siRNA, small interfering RNA; RNAi, RNA interference; RIP, receptor-interacting protein.

To investigate the role of RIP1 in the morphological changes of LoVo cells, the cells were transfected with LoVo-Con siRNA or RIP1 siRNA for 24 h. As illustrated in Fig. 2, knockdown of RIP1 resulted in an apoptotic state and slow growth of the RIP1 siRNA-transfected LoVo cells, with a transition from a typical spindle morphology to a spherical shape. The volume of the cells was reduced, the refractive index was lower than that of normal colon cancer cells and a small number of cells floated on the surface of the medium. The morphological changes of the LoVo cells suggested that treatment with RIP1 siRNA resulted in apoptotic changes.

### RNAi targeting RIP1 inhibits LoVo cellular proliferation

**Figure 3:**
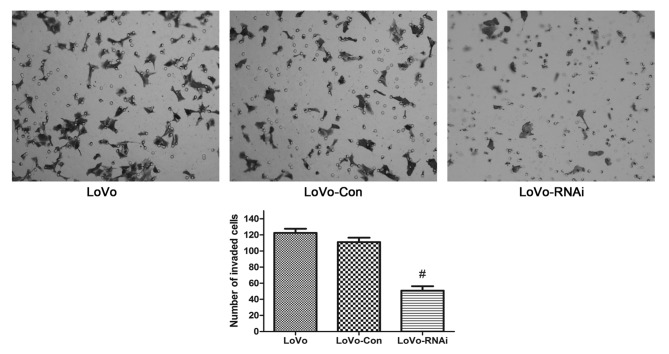
Receptor-interacting protein 1 small interfering RNA decreased the invasive capacity of LoVo cells. LoVo cells, LoVo-Con and LoVo-RNAi cells were loaded into the Matrigel-coated upper chambers of Transwell plates. The cells were counted under a microscope in five random fields at ×200 magnification. ^#^P<0.05, vs. LoVo and LoVo-Con. RNAi, RNA interference.

We investigated whether inhibition of RIP1 affects cell proliferation 24, 48, and 72 h following transfection using an MTT assay. The inhibition of proliferation in the RIP1 siRNA group at 24, 48 and 72 h (36.43±1.23, 59.34±1.74 and 26.34±1.98%) was significantly higher than that in the blank control group (0.00, 0.00, 0.00%) and the LoVo-Con group (26.2±0.36, 26.21±0.90 and 8.38±0.79%). These effects were time-dependent. The results clearly demonstrate that the proliferation of LoVo cells was markedly inhibited at 48 h in the LoVo-RNAi group (Fig. 3).

### Effect of RIP1 siRNA on the cell cycle in human colon cancer LoVo cells

**Figure 4:**
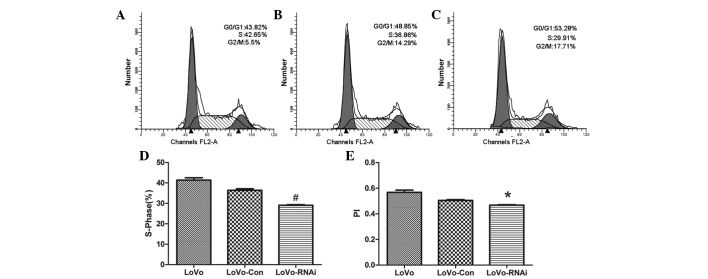
Cell cycle in LoVo cells measured by flow cytometry. The experiment was performed in triplicate. (A) Blank control group; (B) negative control group (LoVo-Con) and (C) experimental group (LoVo-RNAi). (D) Mean S-Phase (%) obtained from LoVo cells. ^#^P<0.05, vs. LoVo and LoVo-Con. (E) Inhibition of the cell cycle in LoVo cells following receptor-interacting protein 1 small interfering RNA transfection (%). The experiment was performed in triplicate. ^*^P<0.05, vs. LoVo and LoVo-Con. RNAi, RNA interference.

To examine the effect of siRNA on the rate of apoptosis in LoVo cells, the treated cells were labeled with PI and analyzed by FCM to detect an apoptotic sub-G0–G1 (M1 value) peak at 24 h following siRNA transfection. It was identified that 53.28% of the RIP1 siRNA cells were in G0/G1 phase and 29.01% were in S phase, compared with the LoVo cells with 43.82% in G0/G1 phase and 48.88% in S phase, and the LoVo negative control cells with 42.65% in G0/G1 phase and 36.86% in S phase (P<0.05; Fig. 4) (16). These data indicate that cell growth inhibition by RIP1 siRNA is associated with cell cycle arrest in G0/G1 phase. siRNA directed against the RIP1 gene appeared to decrease the growth of the cells by regulating the G1 and S checkpoints, and transfection with RIP1 siRNA induced apoptosis in LoVo cells.

### Downregulation of RIP1 decreases the migration of colon cancer cells in vitro

**Figure 5:**
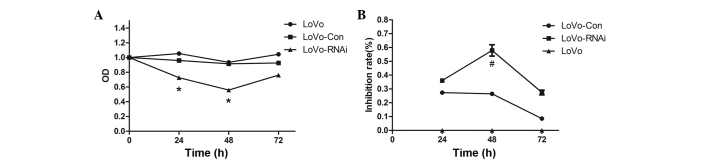
Effects of RIP1 knockdown on cell growth in colon cell lines. The growth was determined using a 3-(4,5-dimethylthiazol-2-yl)-2, 5-diphenyltetrazoliumbromide assay following 0, 24, 48 and 72 h. (A) Absorbance data obtained from LoVo cells. The results are presented as the mean absorbance of the RIP1 cell lines. (B) Inhibition of LoVo cell proliferation at different time points following RIP1 small interfering RNA transfection (%). The SD is shown only for LoVo-RNAi as the other SD values were too low. The experiment was performed in triplicate. ^*^P<0.05 and ^#^P<0.05, vs. LoVo and LoVo-Con group. RNAi, RNA interference; RIP, receptor-interacting protein; OD, optical density; SD, standard deviation.

Cell migration is a critical step in metastasis, and the results demonstrated that RIP1 has a critical role in the metastasic behavior of cancer cells. The effects of RIP1 siRNA on the characteristic of colon cancer cells were examined. Transient transfection of RIP siRNA into LoVo cells resulted in impaired migration capacity compared with the blank control and the LoVo-Con groups, as was evident from the migration assay (Fig. 5). The average number of migrated cells in five fields of view at a magnification of ×200 in the experimental group (LoVo RNAi) was significantly lower than that in the blank control and the LoVo-Con groups (P<0.05). There was no significant difference between the blank control and the LoVo-Con groups (P>0.05).

### RIP1 siRNA inhibits cell proliferation according to the results of the cell counting assay

**Figure 6:**
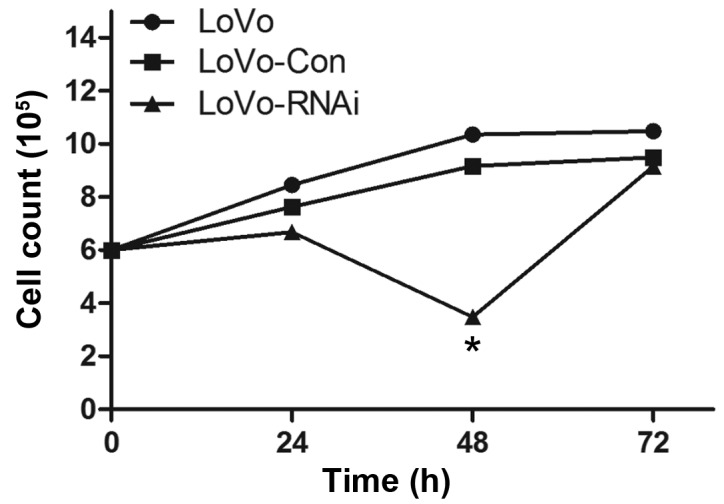
Proliferation of LoVo cells was measured by cell counting assay. The cell proliferation was determined by cell counting 0, 24, 48 and 72 h following transfection. Cell counting data were obtained for LoVo cells. The results are presented as the mean number of RIP1 cell lines at different time points following RIP1 small interfering RNA transfection. The experiment was performed in triplicate. ^*^P<0.05, vs. LoVo and LoVo-Con. RNAi, RNA interference; RIP, receptor-interacting protein.

The number of RIP1 siRNA-transfected LoVo cells following 48 h was 3.33±0.37×10^5^, which was markedly lower than that in the blank control group (10.16±0.25×10^5^) and the negative control group (9.21±0.45×10^5^). There was no significant difference between the blank control and the LoVo-Con groups (P>0.05; Fig. 6).

### Expression of RIP1 proteins in the CRC and normal mucosa

**Figure 7:**
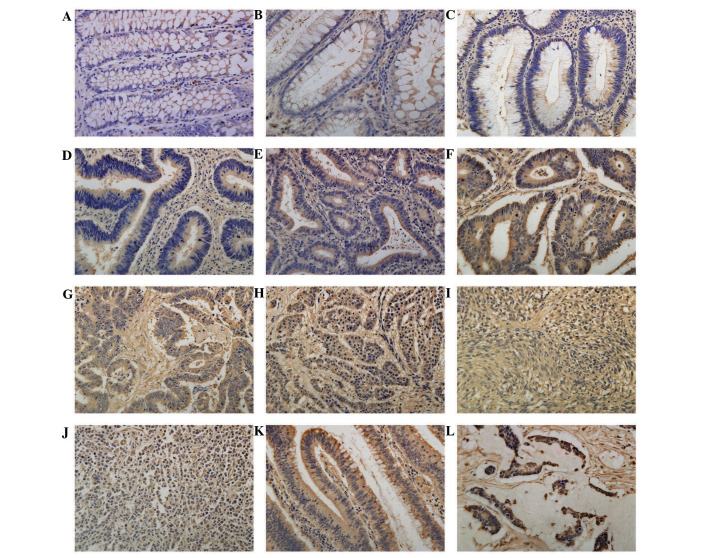
Immunohistochemical staining of colorectal carcinoma tissue sections. (A–C) Immunohistochemical images demonstrated very low levels of RIP1 staining in normal and inflammatory tissues (magnification, ×400). (D and E) The results for RIP1 cytoplasmic staining in colon cancer were weakly positive (magnification, ×400) and (F–L) strongly positive (magnification, ×400). RIP, receptor-interacting protein.

RIP1 protein expression was observed in the cytoplasm. Of the 36 cases examined, positive expression of RIP1 was identified in 2/3 cases (67%) of adenocarcinoma with pathological grade I, 7/10 cases (70%) of adenocarcinoma with pathological grade I–II, 7/9 cases (78%) of adenocarcinoma with pathological grade II, 3/3 cases (100%) of adenocarcinoma with pathological grade II–III, 5/5 cases (100%) of adenocarcinoma with pathological grade III, 2/2 cases (100%) of papillary adenocarcinoma, 3/4 cases (75%) of mucinous adenocarcinoma, 1/1 cases (100%) of stromal tumor and 1/1 cases (100%) of lymphoma. RIP1 was negative in 2/2 samples of (100%) of normal tissues and 7/10 samples (70%) of inflammatory tissues (Fig. 7). The role of RIP1 in the different histological types of colorectal carcinoma may contribute to the degree of tumor differentiation.

### Downregulation of RIP1 expression effectively suppresses RIP1 mRNA expression in colon cancer cells

**Figure 8:**
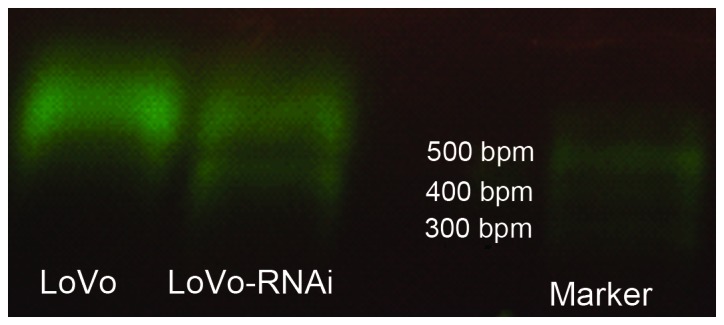
Expression of RIP1 in LoVo human colon cancer cells. qPCR analysis of RIP1 mRNA in LoVo cells and LoVo cells transfected with RIP1-RNAi (LoVo-RNAi). RIP, receptor-interacting protein; RNAi, RNA interference; bpm, binding protection mediated.

To examine the possible roles of RIP1 in colon cancer cells, we knocked down the expression of RIP1 using siRNA. qPCR was performed to examine the effect of siRNA transfection on the RIP1 mRNA expression levels in LoVo cells. Following transfection with RIP1 siRNA, the level of RIP1 mRNA was reduced compared with that of the blank control group (P<0.05; Fig. 8). The results demonstrated that RIP1 siRNA exerted a silencing effect on RIP1 expression *in vitro*.

## Discussion

In 2009, there were a total of 1,479,350 new cases of cancer and 562,340 cancer-related mortalities in the United States. Although the cancer incidence rates have decreased, CRC is second among the three most prevalent types of cancer (lung, prostate and colorectal cancer) in males and the two most prevalent types of cancer (breast and colorectal) in females (17). Progress has been achieved in reducing the incidence of cancer. Currently, fecal occult blood testing, sigmoidoscopy and colonoscopy are used to screen for CRC at an early stage, however the mortality rate is high (18). Despite extensive study, the pathogenesis of CRC remains elusive. Regulatory molecules involved in metastasis are important for the mechanism of tumor dissemination. Cumulative genetic and molecular changes in colorectal tissue induces mucosal instability, premalignant polyps and malignant transformation (19). Therefore, examining the possible mechanism of invasion and metastasis of CRC is important for identifying the molecular targets for clinical treatment.

RIP1 (also called receptor-interacting serine/threonine protein kinase 1, RIPK1) is an essential upstream signaling molecule in TNF-α induced necroptosis (20). RIP1 associates with DRs, including Fas, TNFRI and TRAEL-Rs, that initiate apoptosis. RIP1 is important for T-cell compartment reorganization and TCR-induced activation of the pro-survival NF-κB and MAPK pathways. RIP1 is also significant for B cell development. RIP1-deficient B cells are impaired in NF-κB activation, and RIP1 has an important function in lymphocyte and colorectal carcinoma cell survival and death signaling (21). A number of studies have suggested that RIP1 also has a significant role in tumor metastasis. In 2010, Osborn *et al* reported that the death domain-containing kinase RIP1 was required for necroptosis (22). Furthermore, when RIP1 kinase activity was inhibited, inhibition of necrosis was demonstrated to the proliferative defect caused by FADD knockout. Yang *et al* revealed that RIP1 was modified in cells with damaged DNA and was required for tumor cell survival (23). However, the precise mechanisms underlying the role of RIP1 in tumorigenesis remain unclear.

Kim *et al* reported that CARD6 activates NF-κB as a result of stimulation by RIP1 (24). Among the tissues collected from 103 CRC patients, there were 81 CARD6-positive samples detected by immunohistochemical analysis. This finding suggested that CARD6 may be associated with NF-κB by stimulating RIP1 in colon cancer. Zhao *et al* demonstrated that RIP1 is a key effector for TNF-induced necrosis (25). In human colon adenocarcinoma HT-29 cells, knockdown of RIP1 downstream of MLKL blocked TNF-induced necrosis. HSP70-TRAF2 suppressed the recruitment of RIP1 and inhibits NF-κB activation following stimulation by TNF-α, contributing to the apoptosis in human colon cancer cells (26). In the present study, RNAi was utilized to knockdown RIP1 in LoVo colon cancer cell lines and the biological effects on migration, proliferation, apoptosis, the cell cycle and invasiveness were observed. According to the results, the cells transfected with RIP1 siRNA exhibited morphological alterations from a typical spindle morphology to a spherical shape, suggesting that the LoVo cells may have entered an apoptotic state.

The reduced growth of cells treated with RIP1 siRNA suggested that knockdown of RIP1 by siRNA inhibited the proliferation of colon cancer cells. These data indicate that the RIP1 gene itself may increase the proliferation of colon cancer cells. These results are consistent with a study by Verbrugge and Johnstone that investigated RIP1 in glioblastoma (27).

The results of the MTT assay and flow cytometry revealed that the LoVo cells that were transfected with RIP1 siRNA exhibited slowed growth and an increase in the proportion of cells in G0 to G1 phase. These results suggested that knockdown of RIP1 in LoVo cells effectively inhibits growth and proliferation, and the gene itself may inhibit colon cancer cell apoptosis. Consistent with this data, in a previous study, Handke *et al* proposed a similar phenomenon in viruses (28).

In addition, the results of a Transwell assay also indicated that when the cells were transfected for 48 h, the number of penetrated cells (21±2.731) was significantly lower than that in the blank control group (47±1.238) (P<0.05). Silencing of RIP1 in LoVo cells may significantly inhibit tumor cell invasion and migration. These results suggest that RIP1 may have tumorigenic potential in the LoVo human colon cancer cell line. In breast cancer, RIP1 and NEMO activate the IKK complex and NF-κB to promote tissue-specific migration (29). ANXA1 is required for the recruitment of RIP1 to the IKK complex, and it is important for the activation of NF-κB. ANXA1 overexpression with RIP1 enhances metastasis and reduces survival. Therefore, we hypothesized that following knockdown of RIP1, the invasion and migration capacities of colon cancer cells were inhibited.

In the present study, these data revealed that RIP1 was positively stained in colon cancer. Therefore, RIP1 may be useful for the diagnosis of CRC by immunohistochemical staining. Furthermore, it was demonstrated that RIP1 overexpression was associated with increased metastasis and invasion in LoVo cells. The degree of staining was associated with the TNM staging, and cytoplasmic RIP1 expression in colon cancer was associated with the depth of tumor penetration and cancer stage. The results of the qPCR assay revealed that RIP1 siRNA effectively downregulated the expression of RIP1 and that RIP1 affected the growth behavior of human LoVo cells *in vitro*. Further studies are required to elucidate the precise mechanism of action underlying the effects of RIP1 in colorectal cancer cells.

In conclusion, the present study demonstrated that RIP1 siRNA effectively suppressed RIP1 gene expression in colorectal cancer LoVo cells and regulated malignancy, proliferation, apoptosis, the cell cycle and the invasion capacity of LoVo cells. Therefore, RIP1 may be potential target gene for RNAi in the treatment of CRC, and gene therapy targeting RIP1 represents a novel therapeutic strategy that requires further investigation.
